# Exploration into the opinions of patients with HIV, healthcare professionals and the lay public of the use of microneedles in clinical practice: highlighting the translational potential for their role in HIV infection

**DOI:** 10.1007/s13346-020-00848-8

**Published:** 2020-09-18

**Authors:** Kurtis Moffatt, Caoimhe Quinn, Paul J. McCague, Ryan F. Donnelly

**Affiliations:** School of Pharmacy, Queen’s University Belfast, 97 Lisburn Road, Belfast, BT9 7BL UK

**Keywords:** Microneedles, HIV, Opinions, Healthcare professional, Lay public, Patient

## Abstract

Poor adherence to oral antiretroviral therapy (ART) remains an important challenge in the treatment of HIV. Microneedles (MN) potentially could offer a non-invasive long-acting (LA) delivery approach, avoiding the need for daily dosing of ART. However, this claim has yet to be explored amongst its potential end-users. The aim of this mixed methods study was to investigate the perspectives from various end-users surrounding the translation of MN technology to general clinical practice, with a particular focus on delivery of ART. Quantitative postal questionnaires were distributed amongst healthcare professionals (HCPs) and the lay public (LP). A total of 208 responses were obtained (HCP, 69; LP, 139), with a completion rate of 34.7%. The consensus on MN technology was positive from both demographics (HCP, 97.1%; LP, 98.6%), with further strong support of postulated MN use within HIV (HCP, 97.1%; LP, 98.6%). Qualitative focus groups were employed to investigate in-depth, the perspectives of 12 patients with HIV. Again, consensus on MN technology was positive, highlighting benefits pertinent to HIV, including discreet self-application and potential sustained release thus avoiding daily oral ART and associated side effects. Patient concerns focused on the need for varied MN dosing schedules and a reluctance to change from established ART. The findings of this study provide an initial indication of MN acceptability, particularly for use within HIV, from various end-user demographics. Furthermore, concerns raised advocate the importance of continued translational research in this area and should act as motivators for those in MN development to ensure a patient-centred MN product is delivered.

**Figure:**
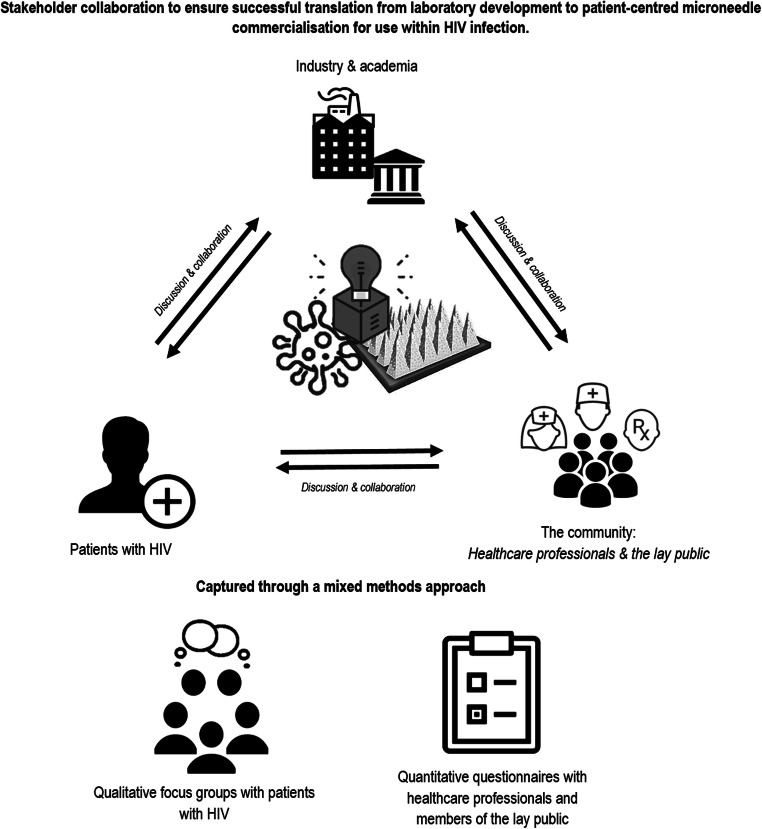
Graphical Abstract

Graphical Abstract

## Introduction

HIV remains a global pandemic with 37.9 million people affected worldwide at the end of 2018. While advances in antiretroviral therapy (ART) treatment efficacy have significantly reduced HIV- and AIDS-related mortality and morbidity, HIV is still progressing at an alarming rate of 1.7 million new cases per annum [1]. Recently, sub-optimal adherence to oral multidrug ART as a result of high pill burden has emerged as the primary cause of treatment failure and development of drug-resistant virus [2]. Accordingly, substantial effort has been invested to reduce dosing frequencies to once-daily oral regimens, while co-formulation has reduced the daily number of pills by offering fixed-dosed combination (FDC) tablets. Nevertheless, treatment fatigue remains a substantial concern.

Long-acting (LA) injectable formulations that permit dosing on a less-frequent basis are becoming an increasingly attractive option to address such adherence challenges [3]. Recently, two LA injectable antiretroviral (ARV) formulations have entered clinical investigation as part of combination therapy administered intramuscularly (IM) at either 4 or 8 weeks, demonstrating non-inferiority to a daily triple-oral ART regimen at week 96 [4]. While this approach may afford some patients the convenience of avoiding daily oral dosing [5], administration by necessity, must be given by IM injection. This poses particular challenge for resource-limited developing countries such as risk of needle-stick injuries, inappropriate reuse of needles and poor disposal practices [6] and raises further concern for needlephobic patients [7]. As such, microneedle (MN) technology presents as a promising alternative platform for delivery of LA ARVs, to not only achieve therapeutic outcomes, but by avoiding both the need for daily pill taking and invasive IM injection [8], should positively benefit patient adherence to therapy.

MNs are minimally invasive devices that painlessly by-pass the outermost later of skin, the *stratum corneum* (*SC*), by forming transient aqueous microchannels thus granting access to the dermal microcirculation located in the inferior skin tissue layers [9], thus increasing the number of potential drug candidates effectively deliverable by the transdermal route. MN consist of needle-like microprojections positioned on a baseplate in various geometries. This novel approach presents numerous potential benefits over conventional methods of delivery in general, however many of which particularly pertinent to that of patients with HIV, offering the potential for controlled release, discrete self-application and a reduction in medication related adverse effects [10]. Furthermore, the prospect of MNs as a potentially viable option for LA treatment or prevention of HIV has been recently supported by a pre-clinical study conducted by McCrudden et al., in which MN-mediated high-dose delivery of an ARV in rat models was successfully demonstrated [11]. Aligning prolonged therapeutic plasma levels exhibited as a result of a single MN application, with the resultant prospect of a reasonable patch size for human use, this study provides substantial evidence to support the future use of MNs as a needle-free alternative of LA ARV delivery. Thus, eradicating the necessity for complicated daily oral dosing, which may provide some patients a convenient approach to manage or prevent HIV. Consequently, it is crucial to consider the perspectives of this key target patient population at the development stage to facilitate translation to a patient-focused MN end-product.

As the evidence base supporting the use of MNs as a successful drug delivery platform continues to grow, the commercialisation of a true MN product has come within reach. However, it is now well recognised in modern healthcare settings that the input of end-user in the early development stage of new interventions cannot be underestimated. Such involvement, or lack thereof, can directly determine the success of a new device or innovation [12]. Whether MN-based products are ultimately a commercial success will depend upon not only their ability to perform as designed but also their acceptability to patients and the clinicians who administer them. Therefore, by engaging with these important stakeholders early in the development process, potential issues can be identified, which allows these factors to be addressed and relevant adaptations to be made if necessary. Previous studies investigating perceptions surrounding MN technology have yielded positive findings with various potential end-user demographics including members of the lay public (LP), various healthcare professionals (HCPs) children and the elderly, being able to identify a number of benefits of MN technology, whilst also highlighting potential suggestions where improvements may be made [13–16]. However, there has been no study to date, that has specifically considered the views and opinions of patients with HIV, surrounding the use of MNs for drug delivery, despite the complexities of current ART. Similarly, no study has extensively considered the perceptions of HCP and the LP of the translation of MN technology to general clinical practice, whilst also highlighting their role in the treatment and prevention of HIV infection.

Therefore, this study utilises a mixed methods approach for the first time, to gain an insight into the views and opinions surrounding future translation of MN technology for drug delivery in clinical practice, with a particular focus on delivery of LA ARVs, from various key end-user perspectives. Quantitative methods (structured questionnaires) were used to investigate the perceptions of various HCPs and members of the LP on a time- and cost-effective large scale, whilst qualitative methods (focus groups) were employed to gain a more in-depth understanding from patients with HIV. With the hope of involving patients with HIV, HCPs and the LP at a relatively early stage, this research aims to act as a motivator to help those involved in MN development to improve and tailor formulation-based research towards translation of a truly patient-centred MN product.

## Methods

### Methodological rationale

Qualitative research designs are often employed in studies involving gathering of knowledge based on human experience relating to a particular topic of interest, thus allowing detailed, in-depth examination of issues [17]. Healthcare typically involves many complex human interactions, rarely described using numerical data or terms, subsequently qualitative methods are well recognised and accepted in healthcare research [18]. However, it is acknowledged that this approach is limited by the lack of generalisability beyond the study sample, and consequently mixed methods studies which combine qualitative and quantitative aspects are becoming increasingly popular, as the combined approach allows exploration of diverse perspectives and uncover relationships that exist between the intricate layers of multifaceted research questions [19]. Furthermore, triangulation of data from more than one collection methods further assures validity of research. As such, the current study adopted a mixed methods approach employing aspects of qualitative and quantitative research designs in two sequential phases. The initial qualitative study consisted of focus groups involving patients with HIV as the subjects. This design was chosen to facilitate viewpoint and idea exploration on this new and complex concept [20] and a lack of previous research on this topic [21]. The preliminary qualitative findings in combination with a review of existing literature also helped to inform the content of the quantitative questionnaire distributed for the second phase of the study. A postal questionnaire was selected to gather the views of HCPs and the LP from across Belfast City and the greater Belfast area. This method of data collection was considered ideal as it allowed the opportunity to more conveniently cover a greater geographical range in a cost effective manner [17]. Additionally, self-administered questionnaires offer respondents a greater flexibility to complete the study, at a time convenient to them, and postal surveys generally attain higher response rates than that of online or electronic surveys [22]. Both study phases were approved under the same protocol by the Research Ethics Committee, School of Pharmacy, Queen’s University Belfast 016PMY2018).

### Phase one: focus groups

**Fig. 1:**
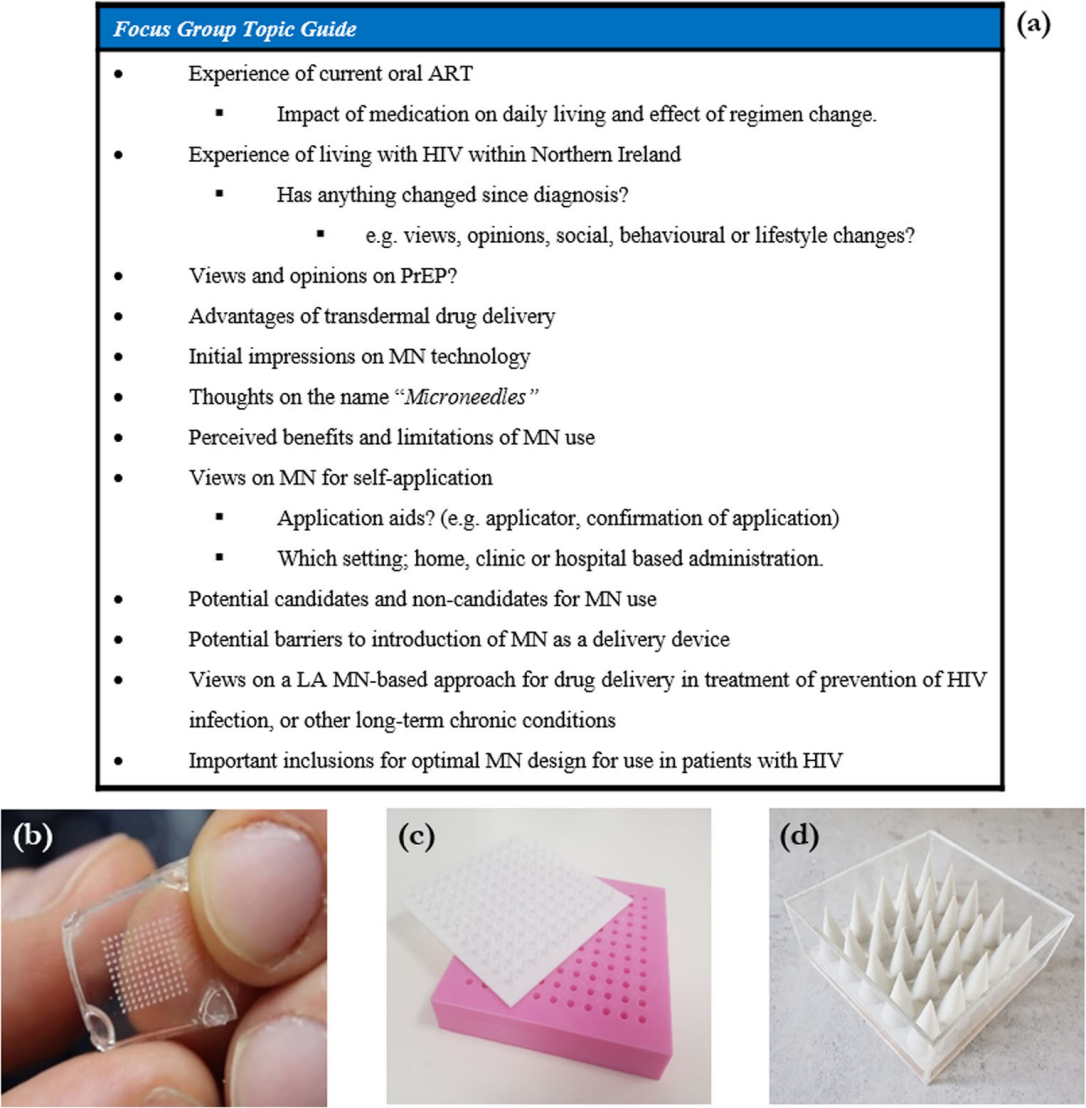
Topic guide for focus group discussion with patients with HIV (**a**); 1 cm^2^ blank polymeric MN array (**b**); oversized MN array skin simulant model (**c**); and oversized MN array contained in a sealed Perspex box (**d**), all used for demonstration purposes

Focus groups were employed to assess the perspectives of patients with HIV on the future clinical translation of MN technology. Focus groups are essentially group based interviews that capitalise upon communication between 4 and 8 participants, in order to generate data [23]. Rather than one-to-one interviews, focus groups were selected, as they permit the collection of views and opinions from a larger number of participants and, the group interaction allow patients with HIV to hear and question others’ opinions and develop or modify their individual standpoints [24]. This qualitative methodology is particularly useful when knowledge on the issue is lacking, which given the novelty of the concept of MN technology, was the context of this study [21]. A topic guide for focus group discussion was developed on the basis of previous studies conducting qualitative-based research investigating MN technologies [14, 15], and then piloted in a simulated focus group with four members of the Clinical and Practice Research Group, Queen’s University Belfast, and based on feedback, the topic guide content was refined to ensure validity. Each focus group began with simple introductory questions pertaining to patient’s current oral ART, common issues encountered and their general experience of living with HIV as a condition, before progressing to the concept of MN and discussion points on the use of such technology as an alternative method of ARV delivery for the prevention and treatment of HIV infection, as summarised in Fig. 1a. To promote discussion, the groups were introduced to the concept of MN technology by means of a short presentation, explaining what they are and their potential role in drug delivery, particularly highlighting their possible use in HIV infection. Blank polymeric sample MN arrays, produced by simple micromoulding processes for experimental purposes (Fig. 1b), were distributed for inspection, as well as two oversized prototype MN arrays, one of which simulated skin insertion (Fig. 1c), and the other was contained in a sealed Perspex box (Fig. 1d). Each focus group concluded with a short summary of the discussion and provided the opportunity for patients to ask any further questions.

### Recruitment of patients with HIV

Non-probability purposive sampling was used to recruit 12 patients with HIV into two focus groups, through a previously established collaborative network with a local HIV support charity. Written informed consent was then obtained from subjects prior to commencing the study. All participants were HIV positive and over 18 years of age.

### Qualitative data handling and analysis

All focus groups were audio-recorded using a digital voice recorder (Sony ICD-PX370, Sony Corporation, Tokyo, Japan). Recruitment and data collection were to cease when data saturation was deemed to have occurred, noted as the appearance of no new themes emerging with subsequent focus groups. However, it was recognised that due to the sensitive nature of the condition under scrutiny, and issues surrounding confidentiality or stigma associated with HIV, that this was likely to be achievable, particularly with relatively small population of interest available (< 160 charity service users). All recordings were transcribed verbatim, and patient identifiers were removed and each participant was allocated a unique identification code to ensure anonymity prior to being imported to NVivo® software (QSR International Pty Ltd., Doncaster, Australia) for thematic analysis. Broad themes were analysed by constant comparison between and within transcripts. As inclusion criteria for the study stipulated that participants were HIV positive and over 18 years of age, this permitted for a wide range of participants from different backgrounds and lifestyles. Therefore, allowing the research question to be explored from various perspectives, a concept known as data triangulation; such an approach has been credited with improving the validity of qualitative research findings [25]. Common themes were then grouped together into subthemes, and following further refinement, core themes, identified to accurately represent the entire data set. Consensus on the emergent themes was reached by discussion amongst the research team (KM, RD, PMcC).

### Phase two: questionnaire

Quantitative structured questionnaires with qualitative aspects (free text responses) were selected to gain an initial broad overview and understanding of the views and opinions of MN technology, from HCP and members of the LP. This approach was selected as it allowed for information to be obtained from a larger sample of participants in a short time period and at a relatively low cost. A self-administered approach was chosen as it avoids social desirability bias known as the ‘interviewer effect’ [17]. A printed postal questionnaire was selected over an electronic survey due to the poor response rates often associated with online questionnaires [22]. In addition, e-mail addresses of HCPs nor the LP were available to the researcher, making electronic distribution unfeasible.

### Sample selection

A pragmatic approach to sampling was adopted. A sample size calculator was used to determine the total number of completed questionnaires required to be representative of that population (https://www.surveysystem.com/sscalc.htm). Assuming an estimated population of Belfast of 340,000 [26], a confidence level and interval of 95% and 5, respectively, the required sample size was 384 respondents. Thus, to obtain a desirable response rate of 60%, 600 questionnaires would require distribution. As such, it was planned for equal distribution of 300 questionnaires to HCP and a further 300 to the LP. While it is difficult to explicitly state a specific sample size for this type of study, larger sample sizes have been recognised as a viable means to maximise questionnaire reliability and have been associated with higher response rates and a reduced non-response bias [27].

### Questionnaire development

A printed postal questionnaire was developed with white background and clear headings as key features of the simple design as these have been demonstrated to increase response rate [28]. The first page of the questionnaire functioned as an information sheet detailing the purpose of the study, and that voluntary consent was implied by questionnaire completion. The second page served to introduce the respondent to the background of MN technology, specifically detailing the technology and explaining its proposed use in drug delivery in comparison with conventional methods of therapeutic delivery. With no MN product currently available on the market, images of MN arrays were displayed in this section to facilitate understanding, and were considered necessary due to the novelty of the concept. The remaining body of the questionnaire was then divided into two main sections: HIV and current ART, MNs in future clinical practice and, demographics (including information about gender, sexuality and whether or not they practiced as a HCP). The questionnaire was designed to ensure more general questions were located at the start and demographics collected at the end, in accordance with previous recommendation [29].

The content of the questionnaire was informed based on a review of existing relevant literature [15, 16, 30], and discussions with the research team and Positive Life NI helped to refine the questionnaire content and structure. Questions were presented in a range of formats including multiple response closed questions with multiple responses (e.g. using a five-point Likert scale ranging from strongly agree to strongly disagree, yes/no/do not know responses and true/false response), in addition to open ended questions enabling free text responses. The inclusion of open-ended questions facilitated the collection of information which may not have been anticipated during questionnaire development [27]. This was considered a likely possibility for the topic under consideration, given the novelty of the concept being explored. Furthermore, the opportunity for free text responses has also been noted to minimise respondent frustration by transferring the control over the responses provided to the participant [27]. The questionnaire was piloted amongst eight volunteers; four members of the Clinical and Practice Research Group, Queen’s University Belfast, who were practicing as pharmacists, and four volunteers from a non-medical background representative of the LP. Those piloted were asked to check for face content validity and stated that no significant issues were encountered. The general feedback provided by these individuals enabled further questionnaire refinement prior to its distribution. The approach undertaken in the questionnaire development aimed to ensure that its content covered the main research questions whilst being relevant to the target audience. The questionnaire where practical was kept as short as possible, with the overall aim of reducing ‘respondent fatigue’ and maximising response rate [17].

### Questionnaire distribution and data collection

Paper copies of the questionnaire were distributed by two methods depending on the greater convenience suited for the target demographic. Postal distribution of questionnaires was utilised for HCPs, in contrast with handout distribution for the LP. The study only targeted HCPs who were currently working in UK clinical practice within the greater Belfast area, and participants were selected at random (www.random.org) from their respective regulatory register (e.g. GMC, NMC, PSNI etc.). Probability sampling was employed to distribute the questionnaire by handout to the LP at various permitted locations in Belfast City Centre (Permit Reference: 448285) on three separate days, and the contact details and addresses of willing respondents noted solely for remailing purposes, which were subsequently destroyed upon completion of the study to ensure anonymity. Following initial stratified sampling, convenience sampling was then employed with each demographic following by sequential snowballing sampling strategies to increase sample size. The questionnaire pack in all cases contained a brief invitation letter detailing information and the purpose of the study, the contact details of the research team, and a freepost-stamped addressed envelope to return the completed questionnaire. The invitation letter was short, reinforced anonymity and clearly highlighted a specific date for completion to maximise response rate [28]. The deadline was set at 1 month following invitation, following the initial distribution, a further reminder was sent to non-responders after 4 weeks to encourage questionnaire completion and return, as reminders are recognised as a facilitator of questionnaire response [28]. The second questionnaire pack contained a reminder letter in addition to a further paper copy of the questionnaire. As the questionnaires were anonymous, it was necessary to mail reminder copies to all respondents in the sampling frame. The reminder letter clearly indicated that those who had already taken the time to complete the questionnaire did not need to complete it again. However, following the first reminder, no further were sent, since an increased number of reminders may irritate respondents and offer little additional benefit [28].

### Quantitative data analysis and handling

Questionnaire responses were collected via postal return and the data entered into SPSS (V 26.0, IBM, Chicago, USA) and GraphPad Prism® V 5.0 (GraphPad Software Inc., San Diego, California) for detailed statistical analysis. Quantitative data was primarily analysed and presented in the form of descriptive statistics. Free text responses for open questions were exported to NVivo® software (QSR International Pty Ltd., Doncaster, Australia) for thematic analysis and collated with Phase One qualitative findings.

## Results

### Phase one: focus groups

A total of 12 (2 female and 10 male) patients with HIV participated in two focus groups from the Positive Life NI (HIV support charity), which provides service to 159 members in total. All participants at the time of the study were > 6 months post-positive diagnosis and currently receiving ART. The mean age of participants was 51 years, and an average of 11.27 years post-positive diagnosis. The median number of ARV medications participants were receiving daily was 2 tablets, whether individual or FDC therapy. The initial focus group consisted of 8 participants whilst the final comprised of 4. All participants were assigned a unique identifier code such as ‘FG1_01’ which indicated this was focus group 1, participant 1. The mean duration of the focus groups was 53 min (49–56 min) and was conducted in Positive Life NI, 20 Derryvolgie Avenue, Belfast.

Following detailed thematic analysis, three core themes were identified, defined as: *Issues with current ARV, Opinions on MN technology* and *Terminology*, and one subtheme defined as: *Perspectives on pre-exposure prophylaxis (PrEP).* Whilst numerous quotations from original transcripts were used to support attitude statements in the formation of the core themes, in order to remain concise, only quotations that expressly summarised the main sentiment have been stated.

### Core theme 1: issues with current ART

The participants reported a complex situation when asked about their current experience of ART or living with HIV as a condition in general. A primary concern of current oral therapy surrounded the regular occurrence of side effects, which ultimately affected the patient’s daily living experience and, consequently, negatively influenced their adherence to therapy. In addition, the majority of participants had experienced ADRs resulting from their ART, which further complicated their health status and, in many cases this lead to complex regimen changes. The continued changes to participants’ complex therapy, such as alteration to the number of tablets, dosing frequency and generic/proprietary switches, caused them confusion and thus their reduced understanding of their ARV therapy. As a result, some participants reported intentional omission of doses or “drug holidays” to avoid experience of such adverse effects. Adherence was found to be complicated further from an unintentional perspective, as many participants stated that at times they simply forgot to take their daily ART, with some stating that in instances of doubt or concern if whether they had taken their ART or not, they would double up on doses to ensure they had the ARV in their system, as it was the ‘… *lesser of two evils* …’ (FG1_03). Whilst participants unanimously agreed with the difficulties and concerns faced with their ART, many stated that it was a “life or death” situation, and that the side effects of the medication were much less a concern than omitting doses:

‘You have to, it’s life and death, we don’t have a choice really’. (FG1_04)

A focal point of discussion surrounded the participants’ experience of living with HIV as a condition and ultimately any stigma experienced and how that impacted upon their daily living. It was obvious from the discussion that all participants had previously experienced or were still experiencing some form of stigma, with the majority of participants in agreement that more education and, consequently, normalisation of the condition is ultimately key to combat such stigma. Some participants even stated feeling ashamed, living in fear of their condition being discovered and, in some instances, went as far to withdraw in order to administer their oral dose:

‘…I would have missed taking my medication in front of friends or family… I was always so scared in case they recognised the tablet or wondered why I left the dinner table …’ (FG2_04)

The majority of participants further agreed that as a result of no noticeable symptoms or visible signs of HIV once controlled by ART; that daily pill taking or clinic visits were generally the only reminder of illness, in which some participants has created to distraction techniques to overcome the psychological aspects of such reminder of their condition in this instance:

‘…I don’t really have any visible effects of this illness…so the medication is just a permanent reminder that I actually have a disease…as soon as I get my medication…I put it into a Centrum® [UK multivitamin] bottle, it looks the same, so in my mind I am just taking a multivitamin…’ (FG2_04)

The discussion of the current ARV medication provision exposed areas where significant improvement could be made, although it also highlighted the ability of those suffering from chronic conditions with complex medication regimes such as patients with HIV to develop coping strategies to manage their medication, remaining resilient and adherent despite the numerous and vast and multifaceted difficulties they reported.

### Subtheme: perspectives on PrEP

As MN technology is postulated for prevention of HIV infection in addition to maintenance treatment, the participants briefly discussed the potential benefits and concerns surrounding of the recent introduction of PrEP, and similar HIV preventative measures. It was discussed that as a result of the use of PrEP, it may in turn promote a potential increase in sexual promiscuity with a corresponding rise in other sexually transmitted infections (STIs) and associated health issues, and, thus, this concern may present as potential barrier to widespread acceptance of PrEP. However, participants ultimately thought this would be a temporary barrier, and with education surrounding PrEP and general sexual health, would aid in its successful implementation. The discussion highlighted valid points from both sides of the debate for the widespread implementation of PrEP, which subsequently may be representative of the wider public perception to such medication. Thus, further reinforcing the need for greater information and education surrounding not only medication but also sexual health service provision. Upon further discussion, the majority of participants largely agreed that the benefits of widespread use of PrEP outweighs the concerns, and consequently, the choice should be available to those who are high risk and require it.

‘To be honest, if someone is going to have unprotected sex, they are going to have it whether there is PrEP or not, so, I think on the plus side it is better that they have PrEP, so at least then they can be protected from HIV…’ (FG1_07)

### Core theme 2: opinions on MN technology

The various potential benefits of MN delivery in comparison with convention means of drug delivery were discussed in detail. Participants initially were quick to highlight the discreet method of MN application, which many stated would have a particular benefit within conditions to which stigma is associated, such as that of HIV. The ability of MN-mediated delivery to circumvent the gastrointestinal (GI) tract and, subsequently, avoid associated side effects that are commonly experienced with conventional oral ART, was stated as a particularly attractive aspect of MN technology. Participants further commented on the minimally invasive method of administration afforded by MNs, which in turn could overcome the well-established limitations associated with conventional hypodermic needle and syringe delivery, such as pain, fear or the need for trained HCP administration, with some recognising that removal of the HCP would further result in a sequential reduction in associated healthcare costs. This engaged further discussion about the possibility for self-administration, and when questioned on the clinical setting they would like to see the device utilised in the future, there was a consensus that as the device offers the potential of self-application, the benefits it affords would be lost in a clinical setting, and unanimously agreed for at home use:

‘Well, one of the main advantages [of MNs] is that you can do it yourself, so why would we go to a clinic, I’d want this at home …’ (FG1_02)

The LA delivery that MNs potentially afford was also recognised as a source of major benefit. It was generally agreed that a patch that could deliver medication for a prolonged period of time would increase adherence to therapy, by reducing pill burden, treatment fatigue and, additionally avoids the need to physically possess or carry medication at all times. Some participants then further stated that, by eliminating the requirement of daily pill taking, it would also remove the constant reminder of illness every day:

‘…the fact you only have to take it once a week, is a good thing, because you are less likely to miss doses, you don’t always need to remember to take them…also there would be a lot less reminder you are being treated… I’d really like to not have that reminder every day that I am ill’. (FG2_03)

Participants were informed that MN currently in pre-clinical studies were envisaged to deliver enough medication for up to either 1 week, a fortnight, or up to a maximum 1 month’s medication after a single application, and explained that with increasing the length of duration of action, would then equate to a proportionate increase in MN patch size. They were then asked to discuss their preference of dosing schedules and any advantages or barriers they could envisage with the schedules. Initially, there was unanimous agreement from all participants that the longer the duration between patch applications was the preferred option:

‘Well if I know its effective and I know I can be confident that it is effective, then why not monthly?’ (FG2_03)

However, as the discussion progressed participants identified possible consequences and drawbacks of longer-acting formulations:

‘… forgetting, yeah, maybe the longer doses would have a downside if we forgot to apply them… we are only human, and only missing one tablet is one tablet… but what if you missed a week or a month worth of medication by not applying a patch’ (FG1_08)

It was also acknowledged the potential for accidental overdose or application by others, and the implications of longer acting formulations may have:

‘…how would you be able to reverse it… like if you applied two long patches, instead of taking one day’s overdose… you now have a week’s or a month’s overdose in you?’ (FG1_08)

The conflict in opinion amongst the participants highlighted that regardless of efficacy or therapeutic outcomes, successful adherence or acceptance will ultimately reside with individual preference and suitability to their lifestyle, and as such, it must be anticipated that not all devices or dosing regimens will be universally accepted.

Interestingly, the benefit afforded by LA MN mediated delivery, in relation to personal safety and welfare in low resource settings was unexpectedly highlighted. Issues synonymous to Zimbabwe, and Sub-Saharan Africa, such as access to medical care, theft, and in more concerning circumstances of which sexual assault and rape occur:

‘… It can be very difficult to access healthcare in Zimbabwe, and if we got these each month and for longer than tablets then it would be really helpful…also when [criminals] get to know that you are taking medication, they can actually come and rob your medication, sell it and… [in some cases] rape you… making HIV spread … they go and sit outside the hospital and they know the department, the minute you are walking out they follow you’. (FG1_01)

Reference was further made to impacted adherence at times when alcohol is consumed recreationally, in which MN patches would overcome issues in these instances:

‘Yeah it would eliminate [the need to remember to take medication] … the only night I would ever forget, very occasionally, to take my tablet is when I have one or two glasses a wine.” (FG1_07)

This triggered deeper conversation about recreational illicit drug use, and the recent growth in ‘*chemsex*’ practices, which is defined as sexual activity engaged in whilst under the influence of stimulant drugs, typically involving several participants [31]. Consequently, those who live such chaotic lifestyles are considered high risk of contracting and/or spreading HIV infection. As such, participants stipulated that these individuals would partake in such activity regardless of consequence; however, MN technology may afford specific benefit in such circumstances:

‘… there are issues surrounding chemsex parties, and people who go to these forget to take their medication, and suddenly become detectable, and they are passing on HIV, whereas if they are taking the patch every two weeks… well realistically, people are going to go to these parties one way or another, so they can… but they are still protected…’ (FG1_07)

Throughout the course of the discussion, aside from the potential future treatment and prevention of HIV, various other clinical applications of MNs were stipulated by the participants, such as diagnostic fluid sampling, alongside potential drug candidates for delivery. As the participants were from non-medical backgrounds, the majority of suggestions were made on the basis of patient own experience with such conditions (e.g. diabetes and epilepsy), other suggestions primarily stemmed from the controlled-release potential that the MN design affords (analgesics) and vaccines were also highlighted due to the avoidance of the use of needles. On the particular basis of avoiding needles as a major benefit of the technology, participants identified children and infants as a specific target population that would make particular benefit of MN technology, due to its ease of access and reduced fear and pain on administration.

Whilst participants highlighted many potential benefits of the technology, particularly in relation to treatment and prevention of HIV infection, they also acknowledged some potential concerns upon its implementation either in conjunction with, or in place of conventional therapy. Initial apprehensions were raised about MN site-specific side effects, such as local effects and sensitivity at the site of application and, further concerns were raised regarding the potential of the risk of infection resulting from breach of the skin barrier. Additional issues pertaining to stock and medication supply that currently affected patients with their oral ART were also discussed as a concern in relation to MN patches, and the possibility of a discontinuation of supply. Some of the participants frequently referred to concerns surrounding the visibility and handling of the patch, such as the resultant implications of losing a patch containing a large supply of medication, and subsequent consequences of someone else handling it:

‘…what if you lost the patch or dropped it under something? You lose a tablet, that’s one tablet gone…but that’s an entire course of treatment lost that would worry me…or what happened if someone picked up the needles would the drug go into them?’ (FG1_05)

These comments provoked further discussion surrounding the handling of the device and, alongside the design of the patch itself, participants raised issues surrounding the product packaging, in which design consideration should take into account child safety, with child resistant caps available on oral medicine bottles cited as an example. However, some participants opposed this idea, as they personally suffered from reduced dexterity, which should be considered for the target consumer in the packaging of such devices:

‘…the problem with childproofing is that older people with arthritis have trouble opening them up’. (FG1_02)

Previous concerns associated with failing to remember to apply MN patches over longer periods were also discussed with particular regard to the product packaging. Some participants suggested packaging adjustments as memory aids, with numbering and dating of the patches, such as those available for tablets in the form of calendar packs, whilst other participants suggested the use of mobile phone applications and adherence aids.

The concept of the device’s novelty was discussed further, in which concerns were raised over the limited number of conditions, at least initially, MN patches would be available to treat when they reached market. The concept of combining multiple medications into a single MN patch and its feasibility was questioned, as many participants were on numerous medications for various conditions. This then lead some to query just how beneficial replacing a single therapy would be for patients, which may in contrast complicate an already complex dosing regimen:

‘I can see the problem being that if someone still had to take tablets for somethings, so they are still taking a tablet every day, but then they have to remember to take a patch every two weeks, and they have to take an injection every… it’s a lot to keep in mind’. (FG1_07)

Following a brief explanation and demonstration by the researcher, participants thought the application of a MN patch appeared to be relatively simple, some apprehension was made however as to confidence to when the MN would be correctly applied, or as to when the dose had been delivered:

‘Yeah, something to think about… has it gone in or has it not… whereas my tablet goes down my throat, I know it’s in there…’ (FG2_02)

Some queried the pressure that would need to be applied in a reproducible manner, and stating the preference for use of an applicator to aid in administration, with reference made to similar devices used for insulin in diabetic therapy, or adrenaline within anaphylaxis as example based upon their personal experience. However, others stated that whilst they would feel confident to apply the patch independently, in the instances with something as significant and unforgiving as ARVs, they would at least want some form of positive feedback mechanism, designed to respond to correct application with or without an applicator, with some suggesting a colour change, which was met with unanimous approval from the participants. There was also generally an agreement that upon first use of an MN, as with any medical device, training and verification by a HCP would be required to instil confidence in their application technique, with a few of the participants again drawing from personal experience of similar medical devices:

‘I’d like to see it in front of… the first time, maybe on one of my visits [to the clinic]… you know you put it on but there’s someone there to make sure you are doing it the right way? I already use those Freestyle® Libre sensors, and I change them myself, so I’d be happy to, once I’ve seen it once’. (FG2_01)

Despite expressing favourable opinions surrounding MN technology, there was a consensus that those who have been established on ART, would be reluctant to change, particularly when their current treatment is adequately controlling their condition, and thus would find it difficult to break their accepted conventional routine to such a novel innovation:

‘…why would you want to risk changing if you are on something that works’ (FG2_04)

This reluctance to change was further reinforced by the participants when asked for their preference in method of future ARV delivery, if given the choice of oral tablets, IM injection or MN mediated delivery. Despite the benefits that either LA IM or LA MN-mediated delivery stands to potentially afford, there was still a consensus that oral delivery would still be the preferred method of ARV administration.

‘…tablets…because yes, you can physically feel it in your mouth and going down your throat, you now you’ve taken it… you get some reassurance from taking the tablets daily’. (FG1_05)

However, when asked about specifically about preference of the two methods of LA ARV delivery (IM or MN), there was unanimous agreement in favour of self-administered MN application rather than monthly HCP administered IM injections.

‘No, I wouldn’t like an intramuscular injection at all, no. I would rather a microneedle definitely… I go to the clinic once every 6 months, and that’s more than enough for me’. (FG2_03)

Interestingly, in contradiction with their previous statements, participants who previously displayed the greatest reluctance to change from current ART, did however state that if the technology was commonplace within healthcare settings, and thus no longer considered what they thought as ‘experimental’, then they would express a willingness to try. Therefore, a potential barrier to patient acceptance may be highlighted as the foreign nature and novelty of the device. Some went on to highlight that this reluctance to change was not solely linked to MN technology, but to the introduction of any innovative medical device in place of conventional therapy. Thus, it was recognised that appropriate patient education and counselling is central to device acceptance and introduction. Moreover, despite expressing their own individual reluctance to change, there was a general recognition that treatment naïve patients or those of a younger generation would be much more accepting of MN technology, and thus stand to make avail of the benefits the innovation affords:

‘I think the new guys… might like the idea, but for us you know that have had a few years… we like what we like…’ (FG1_04)

Despite inconsistencies within various aspects of the discussion, the participants’ final overall impression of the technology was positive. The general feeling of the participants regarding clinical implementation of MN technology over traditional therapy was summed up by the following quote:

‘If that was the only thing you were on, or were ever on, then brilliant’. (FG1_06)

### Core theme 3: terminology

The terminology surrounding MN technology, which initially began as brief discussion, soon became a major focal point of debate between participants. There was hesitation as to the inclusion of ‘needle’ within the title, with the assumption that this would lead people to expect a needle within the conventional sense, and with that, the negative connotations of hypodermic needles, creating a potential barrier to patient acceptance of MNs as a potential delivery device

‘It’s misleading… some people don’t like anything associated with needles… and it has needle in the title… that will need some explanation’. (FG1_03)

Alternative names were suggested by the participants based on the appearance or potential use of the device including; ‘invisipatch’, ‘microstars’, ‘meddiss’, ‘microdots’, ‘microdarts’, ‘transdermal plus’ or ‘transdermal extra’. despite extensive discussion, no agreement was reached as to what the device should be referred to; simply that some firmly believed that ‘needles’ should be removed from terminology associated with the device. Others however were in favour of the term ‘microneedles’, failing to see the significance of the name of the device, accepting it as an ‘accurate descriptor of the device’ (FG2_01), and that the impact of the name could be minimised with a brief explanation and counselling by a HCP of what exactly MNs were. Recently, the World Health Organisation (WHO) have also suggested removal of needles entirely from terminology associated with the device, and replaced with ‘array’. Unlike the divided opinion surrounding the name microneedles, the novel suggestion of ‘Microarray patches’, was met with unanimous disclaim from participants in both focus groups, stating that it sounded ‘… digital… like a circuit board’. (FG1_06), or ‘… sci-fi… like Star Trek’. (FG2_03). Consequently, it was then suggested that the more technical the terminology of the device becomes, it may in turn potentially further implicate patient acceptance, as the device initially may seem more daunting than what it actually is.

### Phase two: questionnaire

#### Respondent characteristics

A total 600 questionnaires were distributed equally amongst 300 HCPs and 300 members of the LP. A total of 208 completed responses were obtained (HCP, 69; LP, 139), with a response rate of 34.7% (HCP, 23%; LP, 46.3%). All responses returned were considered complete, with no questions left blank or unanswered. Of the total HCP respondents, those involved included doctors (42%), nurses (17%), pharmacists (39%) and other (2%). In the instances where there was no visible difference in the trend of two demographic (HCP and LP) responses, they were presented collectively. Respondent demographic characteristics are summarised in Table 1.

**Table 1:** Demographic characteristics of HCP and LP respondents to questionnaire

Demoraphic	HCP (%)	LP (%)
Gender		
Male	42	34.50
Female	58	64.70
Trans-person	–	0.70
Age (years)		
18–24	5.80	61.90
25–34	43.50	12.90
35–44	20.30	5.00
45–54	23.20	12.90
55–64	5.80	3.60
65+	–	3.60
Prefer not to answer	1.40	=
Sexuality		
Heterosexual	93.50	95.70
Homosexual	4.30	2.90
Bisexual	1.40	1.40
Prefer not to answer	0.7	–
Education		
Secondary	–	14.40
Tertiary	–	10.10
Higher	100	74.80
Prefer not to answer	–	0.70
Ethnicity		
White British	50.70	52.50
White Irish	29.00	18.70
African	–	1.40
African American	1.40	–
Arabic	1.40	–
Chinese	1.40	9.30
Indian	–	4.30
Indonesian	1.40	–
European British	1.40	–
Prefer not to answer	11.60	13.70

#### Questionnaire responses

**Fig. 2:**
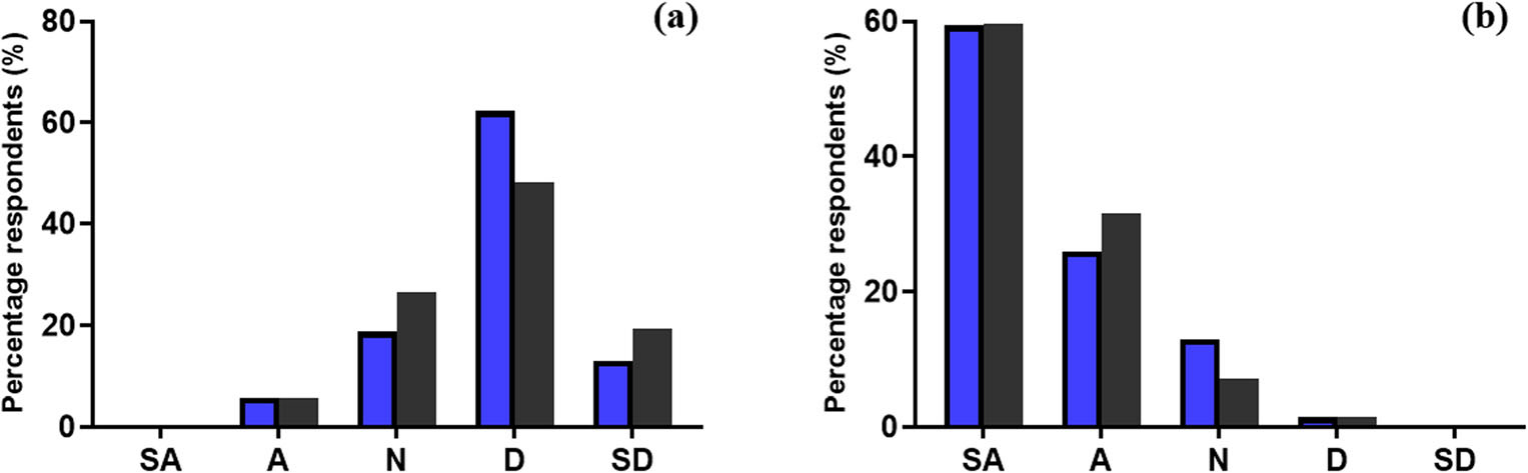
Percentage of HCPs (*blue bars*) and members of the LP (*grey bars*) respondents who strongly agreed, agreed, neither agreed or disagreed, disagreed or strongly disagreed with the two following research statements: ‘There is a high level of education surrounding the topic of HIV and/or AIDS within Northern Ireland.’ (**a**) and ‘Medication (PrEP) that reduces the risk of HIV transmission should be available on prescription within Northern Ireland.’ (**b**), respectively

Respondents were initially invited to state their level of agreement with the statement: ‘There is a high level of education surrounding the topic of HIV and/or AIDS within Northern Ireland’. The responses are represented in Fig. 2a. Similar trends in response were observed from both HCP and LP, with a high level of disagreement (D and SD) noted from both HCP (75.3%) and LP (67.6%). Respondents were then asked if they were previously aware of PrEP, proceeded by asking their level of agreement to the statement: ‘Medication (PrEP) that reduces the risk of HIV transmission should be available on prescription within Northern Ireland’. The responses are illustrated in Fig. 2b. It was revealed that of the respondents, 82.6% of HCP and 37.4% of LP were previously aware of PrEP. HCP awareness was expectedly higher due to their education in medicine. Despite a large gap in awareness of the preventative medication, similar trends in responses were noted again, with a high level of agreement (SA and A) from both respondent groups that PrEP should be available on prescription for those who require it, with 85.4% of HCP and 91.3% of LP positively supporting its prescribed use, respectively.

Prior to investigating the views and opinions of HCPs and the LP regarding MN technology, the respondents were asked: ‘Have you previously heard about microneedles?’ Of the respondents, 72.5% of HCPs were previously aware of the technology, whereas only 27.5% of the LP had heard of MNs. This gap in the MN device awareness actually provides benefit to the topic under investigation, as by exploring the combined views of various perspective sources provides a more complete picture of the proposed research question under investigation. Based on their previous knowledge, or what they had read from the brief introductory information provided with the questionnaire, respondents were then asked what they perceived were the benefits of MN technology in comparison with conventional or traditional methods of delivery currently available on the market. Both respondent demographics displayed similar trends similar in their level of agreement in responses to the posed individual questions, as such their collective responses are documented in Table 2.

**Table 2:** Collective respondents’ initial impressions of the benefits of MN technology. Percentage of collective (HCP and LP) respondents who strongly agreed, agreed, neither agreed or disagreed, disagreed or strongly disagreed with the listed potential benefit of MN technology

Potential benefit compared with conventional methods of administration	Strongly agree	Agree	Neither	Disagree	Strongly disagree
Reduced pain on administration compared with injection	62.5	34.6	2.9	0.0	0.0
Alternative to oral medicine	48.1	41.8	5.8	3.4	1.0
Potential for controlled release (meaning less-frequent dosing)	54.3	41.8	2.9	1.0	0.0
Ability to self-apply with minimal training	57.2	38.0	3.4	1.4	0.0
More discreet method for delivery	48.6	38.5	10.6	2.4	0.0
Reduced fear compared with injection	61.5	33.2	4.3	1.0	0.0
Reduced needle stick injuries	62.5	30.3	6.7	0.5	0.0
Good for children	53.8	33.2	9.1	3.4	0.5
Good for elderly	51.4	39.4	7.2	1.9	0.0
Good for needlephobes	61.1	32.7	4.8	1.0	0.5
Good for diabetes	52.9	34.6	10.6	1.9	0.0
Good for long-term conditions (taking many tablets)	54.3	37.0	7.2	1.0	0.5

As shown, respondents recognised many of the potential benefits of MN technology compared with their conventional counterparts, showing high levels of agreement (> 85%) to all postulated advantages of the technology. In contrast, respondents displayed very little disagreement (< 5%) or opposition to the any of the stated potential benefits.

Respondents further suggested other potential benefits of MNs beyond those listed, some of their suggestions included: no requirement of a HCP for administration, discreet application, blood/fluid sampling for diagnostic purposes, suitable to those with chaotic/unstable lifestyles, ease of storage/user friendly, less risk of diversion, reduced bill burden, reversible in the event of accidental application, advantageous in developing/low resource countries and, lastly, the potential for rapid distribution in the event of vaccination emergency.

The terminology surrounding MNs was then investigated, asking respondents their opinion on the name of the device, as previously some have found the terminology misleading or off-putting [15]. Both HCP (86.9%) and LP (88.5%) reported a favour of the term microneedle as the name of the delivery device, with some stating, ‘it accurately describes what the device is’. In the cases where respondents disliked the terminology microneedles, it was due to the association of the word needles in the title, and while they did not always suggest alternatives, they stated that this would need removed in order to make the device more appealing, due to negative connotations associated with conventional injections. They also stated that the alternate name proposed by the WHO, ‘Microarray patch’, was not appealing as it sounded ‘electric’ or ‘digital’ rather than medical. Additional suggestions that were stated by respondents included ‘Permeation enhancing patch’ or ‘PEP’ for short. However, due to use of this abbreviation already within post-exposure prophylaxis of HIV, this was deemed unsuitable. More frequently stated suggestions also included micropatch and ‘microdermal patch’.

Respondents were then invited to state their level of agreement with concerns that had been previously raised in relation to MN technology. This follows on from a 2011 study conducted by Birchall et al. [16], in which participants highlighted various reservations or issues regarding the use of MNs as delivery devices. However, since then, studies have since addressed such concerns, and as this substantial evidence base now rapidly progresses MN technology closer towards commercialisation, than compared with almost 10 years ago, it was necessary to investigate if these concerns still pose a potential issue with eventual end-users. Existence of such concerns may present potential barriers to the successful implementation of MN technology as accepted therapy in place of conventional methods. The respondents’ collective responses to such previously stated concerns are documented in Table 3.

**Table 3:** Collective respondents’ concerns. Percentage of collective (HCP and LP) respondents who strongly agreed, agreed, neither agreed or disagreed, disagreed or strongly disagreed with the previously stated concerns raised in literature regarding MN technology [16]

Question	Strongly agree	Agree	Neither	Disagree	Strongly disagree
How would you know if the drug has been delivered?	15.9	50.5	12.5	15.9	5.3
Is the delivery as fast as oral or hypodermic needle delivery? Is there a delayed onset?	12.5	43.3	16.8	20.7	6.7
Are microneedles expensive to produce?	14.4	31.3	16.8	24.5	13.0
Is there a potential for misuse and abuse, for example, by drug-users?	13.5	29.8	17.8	24.5	14.4
Is there a potential for cross contamination (i.e. microneedle reuse and transfer of pathogens e.g. bacteria)?	14.9	39.4	13.0	18.3	14.4
Different people have variable skin thickness, would this affect application of the microneedle?	16.8	39.4	14.9	18.8	10.1
Is there difficulty in delivering a very small or very large volume of drug?	13.5	46.6	18.8	14.9	6.3
Is there an increased risk of infection after applying a microneedle?	7.2	34.6	20.2	24.0	13.9
Would funding microneedle research, take away from funding other NHS treatments?	8.2	19.2	24.0	27.9	20.7
In an emergency, would a microneedle be capable of delivering the drug as quickly as a hypodermic injection, regardless if it caused pain or not?	33.7	43.8	9.6	8.7	4.3

Despite previously high levels of agreement and recognition of benefits that MN technology stands to afford, when presented with previous concerns from the study by Birchall et al. [16], respondents still expressed the same uncertainties surrounding the technology. Respondents from both HCP and LP showed more agreement with the concerns than disagreement, with the exception of NHS funding to which they would be less concerned with this issue (48.6%). Respondents additionally stated further reservations regarding the device in their free text responses, which echoed the concerns previously raised in the focus group study. These included the potential requirement of safe disposal facilities, the level of dexterity required for self-application in the elderly, and the risk of local sensitivity reactions at the site of application.

Based on their current understanding and awareness, respondents were then asked specifically if they believed that MN technology would provide particular benefit in the treatment and prevention of HIV infection. There was unanimous agreement from both respondent groups that MNs would provide particular benefit in the treatment and prevention of HIV infection, with 97.1% of HCPs and 98.6% of the LP expressing their agreement, thus showing considerable support for potential future MN implementation to ART.

**Fig. 3:**
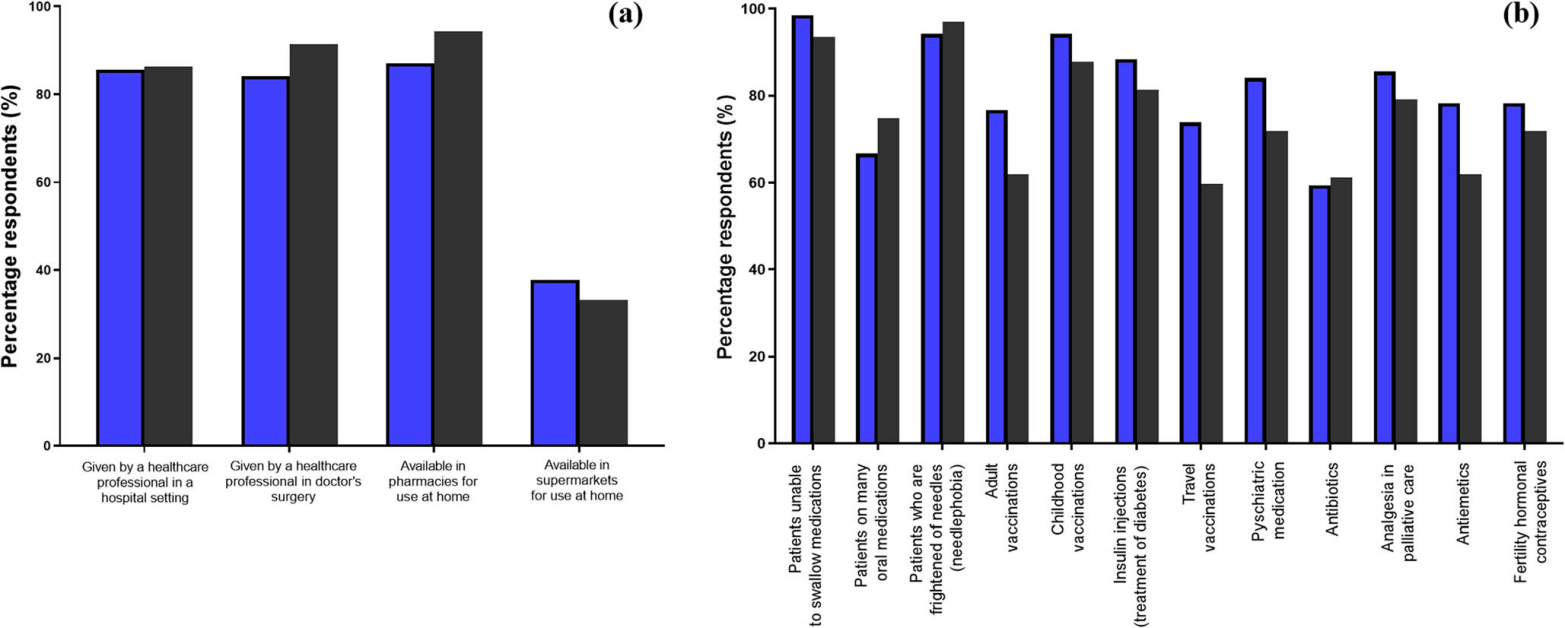
Percentage of HCPs (*blue bars*) and members of the LP (*grey bars*) respondents who selected the underlying option when asked the following research questions: ‘Where would you like to see microneedles used in future practice? (Please tick all that apply)’ (**a**) and ‘In which of the following situations do you think microneedles would be particularly useful? (Please tick all that apply)’ (**b**), respectively

As a major postulated benefit of MN technology is the potential of self-application, respondents were asked if they believed they could confidently apply the device at home by themselves, and further asked to document anything that they believe would aid in reassurance of successful application. Both respondent demographics displayed high levels of confidence in their ability to self-apply a MN device at home (HCP, 89.9%; LP, 86.3%), without the need of a HCP or an application aid. In cases where respondents stated they would not feel confident to apply MN patches themselves, they generally stated in favour of inclusion of a positive feedback mechanism, with the majority preferring a colour change indicator that highlighted when the correct amount of pressure had been applied. Along with this, respondents frequently stated the need for training by a HCP, at least initially, was essential to instil confidence in the correct technique or, in the absence of a HCP, an official online video guide demonstration. In either case, some respondents stated that a clinic review would be beneficial to ensure technique was accurate. The incorporation of a patient-information leaflet (PIL) was stated less often than the latter options. In further evaluation of the support of self-administration, respondents were invited to indicate which setting they would like to see MNs used in future practice. Respondents were asked to tick all options that applied. The responses are illustrated in Fig. 3a. Respondents from both demographics indicated favourable implementation of the device for commonplace use in healthcare settings, such as a hospital (HCP, 85.5%; LP, 86.33%) or a doctor’s surgery (HCP, 84.1%; LP, 91.4%), and with the highest indicated choice, that the device be available within the community pharmacy for use at home (HCP, 87.0%; LP, 94.3%). However, only 37.7% of HCPs and 33.1% of LP selected availability within a supermarket. Respondent were then further asked to provide their views on the potential futures uses of MNs, provided with a number of potential options, which had been previously identified in literature by similar demographics [16]. The respondents were asked to tick all that applied and provided free text response space to state any other potential uses not stated in the questionnaire. The responses are shown in Fig. 3b.

Interestingly, despite the different level of medical knowledge, responses were essentially similar between HCPs and the LP, indicating the suitability of MN technology to be applied in all the options stated (> 60%), with only a few noticeable exceptions, that HCPs felt a greater advantage of MN in the use of adult and travel vaccinations, antiemetics and psychiatrics than the LP. Respondents then suggested any further potential drugs, drug classes or conditions that could potentially benefit by MN-mediated administration that were not already stipulated within the questionnaire, and their free text responses are documented in Table 4.

**Table 4:** Potential future clinical uses of MN as stated by respondents in free text space

Potential drugs, drug classes or conditions for administration using MNs			
Alzheimer’s	Reduced dexterity	Localised cancer	Tuberculosis
Dementia	Neonatal delivery	Trancheostomy patients	Veterinary administration
Autism/easily distressed patients	Rheumatoid arthritis	Analgesics/opioids	Epilepsy
Nicotine replacement therapy	Antihistamines	Stroke patients (Nil By Mouth)	Warts, fungal nail or localised skin conditions
Heparin (anticoagulants)	Dental injections/anaesthesia	Adrenaline/Epipen®	Malaria

**Fig. 4:**
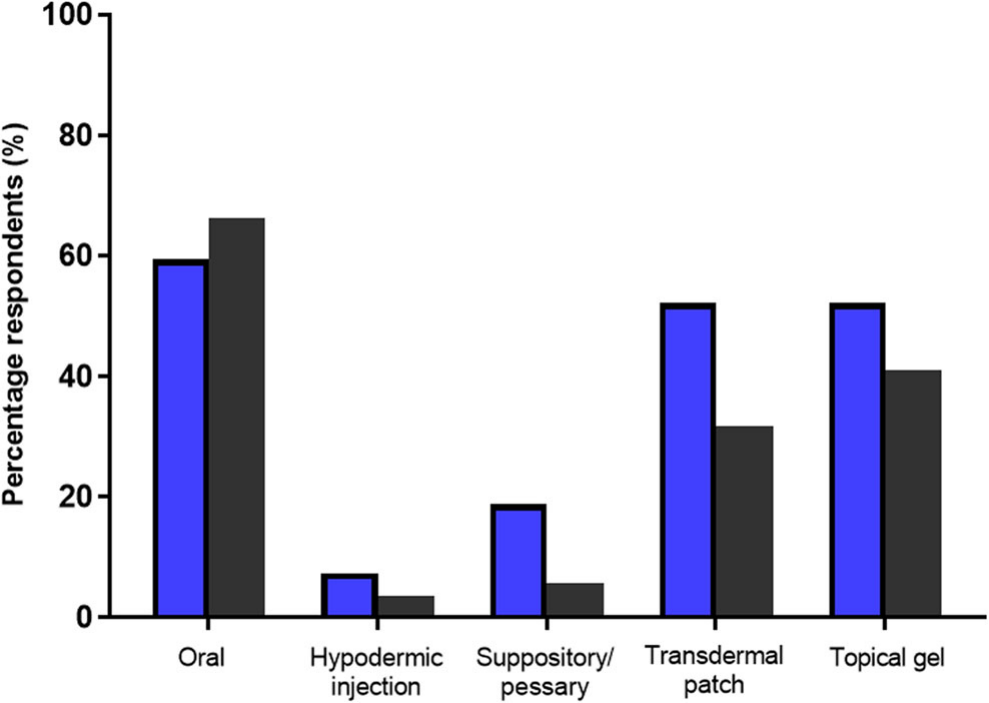
Percentage of HCPs (*blue bars*) and members of the LP (*grey bars*) respondents who selected the underlying option in preference to a MN patch, when asked the following question: ‘If given the choice for delivering the same medicine, would you prefer the stated conventional delivery method, or a microneedle patch?’

Respondesnts were then asked to indicate their preference if given the choice of delivery of the same medication, would they prefer the stated conventional methods of delivery, or a novel MN delivery system. Again, HCP and LP indicated similar responses, as such, their combined response to the question is reported in Fig. 4.

**Fig. 5:**
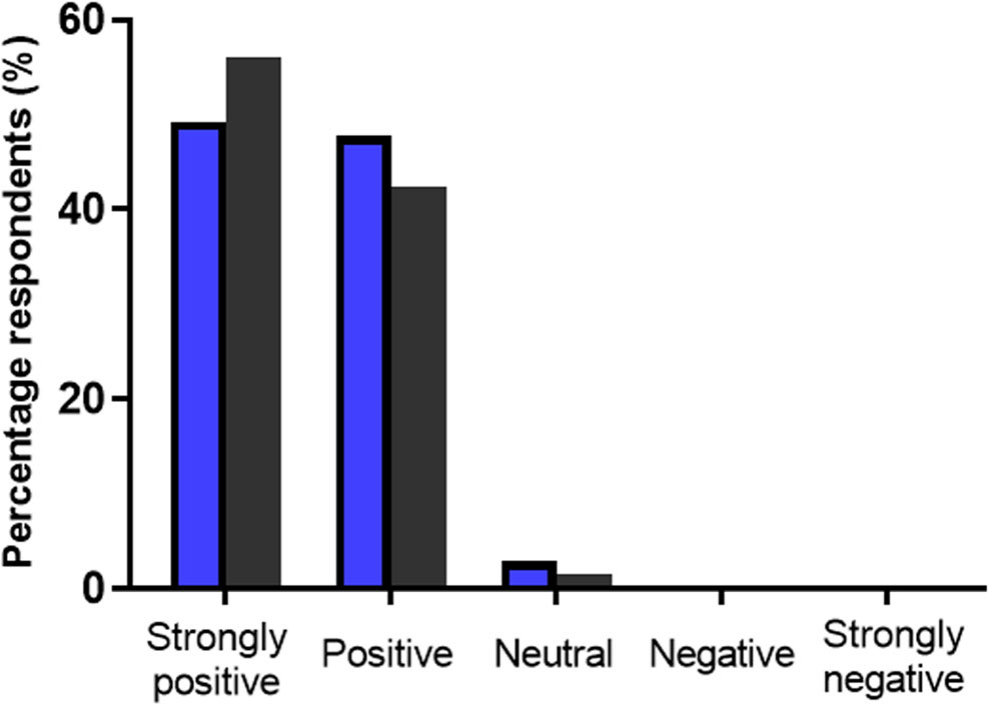
Percentage of HCPs (*blue bars*) and members of the LP (*grey bars*) respondents who selected the underlying option when asked the following question: ‘Based on your previous knowledge, and the brief introduction to microneedle technology provided, what is your overall opinion of the delivery device? (Please tick one option only)

Despite preferable use of MNs highlighted in various circumstances, as illustrated by the overall trend in Fig. 4, HCPs were somewhat more hesitant than that of the LP to side in preference of MN-mediated delivery over stated conventional methods. However, it is perhaps understandable that HCPs may prefer well-practiced and proven delivery methods in favour of novel non-commercialised technology, particularly for conventional methods that possess similar routes of administration to that of MN-mediated delivery. This was demonstrated by the choice of transdermal patches and topical gels, respectively, in which a minority of only 47.8% HCPs in each circumstance selected MN-mediated delivery in preference of the stated conventional means. Reassuringly, however, the greatest preference for MN-mediated delivery was shown in favour of conventional hypodermic injection by both demographics (HCP, 92.7%; LP, 96.4%). In contrast, oral delivery as expected was revealed to still be the preferred method of medication administration, with only 40.6 and 33.8% of HCP and the LP, respectively, preferring a MN patch to a conventional oral tablet. Upon reflection however, it is also possible that due to the novelty of the MN concept, coupled with no MN product currently on the market for comparison, both demographics may not have sufficient experimental knowledge on the background of MN technology to make an informed decision on this particular question. Lastly, respondents were invited to provide their overall impressions of MN technology as a novel alternative method of delivery, indicating their feelings whether positive or negative of the delivery device. Figure 5 illustrates the respondents’ final response.

The overall response to MN technology from respondent demographics was positive, with 97.1% of HCPs, and 98.6% of the LP indicating positive overall impressions, of which 49.27 and 56.2% were strongly positive, respectively. No respondents indicated an overall negative impression of the technology.

## Discussion

### Phase one: focus groups

The use of current oral ART in patients with HIV presents a unique set of challenges, due to a complex interaction of factors largely implemented by the requirement for life-long daily adherence. The problems initially described were in regard to treatment itself, often agreeing that the high pill burden easily leads to confusion surrounding prescribed treatment [10]. Reports of commonly experienced side effects and ADRs often lead to changes in prescribed therapy with altered posology, further resulting in a loss of understanding [32]. Unfortunately, the unique experience of stigma still remains an ongoing challenge, leading patients to exhibit drug holidays as a result of fear of diagnosis discovery, self-shame or simply to remove the reminder of illness [33, 34]. Notwithstanding, participants stated at times they simply forgot to take their ARVs, which is recognised as the most common reason for unintentional non-adherence [35]. Furthermore, a good patient-healthcare-provider relationship revealed to be an invaluable motivating factor in attaining optimal adherence through informed, non-judgemental communication, establishing realistic expectations [36]. Many of these factors where recognised as potential barriers to future implementation of PrEP [37], with additional concerns raised surrounding widespread use promoting careless sexual promiscuity [38]. Education and public engagement were revealed to be key to addressing such attitudes of not only PrEP, but surrounding HIV in general, and highlighted a need for greater public awareness and involvement in healthcare transformation prior to novel interventions [39].

The favourable attitudes patients exhibited towards MN technology echoed that of previous studies investigating the acceptability of MNs as a drug delivery platform, with patients quick to note that removal of pain, fear, the need trained administration, and particularly relevant in this case, the reduce risk of blood borne transmission that is associated with conventional IM injection were primary advantages afforded by MN technology [14–16]. Subsequently, patients expressed a clear preference of the future implementation of a MN-mediated ARV delivery system in favour of postulated IM LA ARV treatment in clinical development [4]. It follows that there was apprehension surrounding the term needle in the terminology of the device, a common concern in previous studies [15]. While some agreed with appropriate education it was an accurate device descriptor, others thought all association should be removed from the device, particularly if anticipated to avail benefit within children and needlephobic patients [7, 40]. Patients identified that well-established benefits of MN technology would be particularly pertinent to overcome current issues with current ART, such as avoidance of GI-related side effects [41]. Previously, the potential of MN self-application with minimal training has been demonstrated [42], which was acknowledged by patients as particularly attractive for use within HIV treatment for numerous reasons, including that the discreet manner of application was deemed a major benefit for conditions in which stigma is prevalent [34]. Moreover, that postulated self-use of the device at home could potentially reduce the frequency of clinic visits, and by further removing the requirement of a HCP for administration, mutually reducing treatment costs [11]. Patients interestingly recognised the potential controlled release afforded by MN-mediated delivery, which was relatively unexplored in qualitative studies until now. LA transdermal patches have been an attractive approach with patients for other drug candidates, such as buprenorphine which is delivered over a 7-day period, shown to significantly improve adherence to therapy [43]. Patients acknowledged that a LA transdermal approach would avoid the need daily oral dosing, thus removing the potential to unintentionally forget to take their ART, and further removing the need to keep and transport medications as they undertake their daily activities. Some stating particularly relevance for instances in which alcohol consumption or chaotic lifestyle choices impair memory [44]. Furthermore, by removing the need to take medications daily, reduce the impact of stigma for a particular patient, as the constant reminder of the illness would be reduced, and by their own admission, they would not have to withdraw from present company to take their medication in private. However, patients expressed varied opinions as the to the preferred as to the exact duration of this LA patch, which may present a challenge for those in MN development, as regardless of a products efficacy it may never be truly universally accepted, and thus industry must strive to meets the needs of the individual patient end-users.

Well-characterised concerns surrounding MN technology such as the potential for skin allergy sensitisation, questions of safe disposal and risk of local infection were once again highlighted by patients [13], and as commonly expressed in previous studies, patients reinforced the need for some form of assurance that either the drug had been delivered or some form of confidence in their application technique [14, 15]. Patients initially suggested pen-type applicators [45]; however, others were in favour of manual application by hand, following initial instruction by a HCP and a take-home PIL [42]. Participants responded very positively to the inclusion a positive feedback mechanism such as a pressure responsive or colour change pad to inform them when the device had been correctly applied, echoing that of previous studies [15]. Concerns were further raised in relation to the eventual size of MN patches, and if they would be too large for self-application; however, in a recent study by Ripolin et al. (2017), it was demonstrated that MNs of a relatively large size (16 cm^2^) could be successfully applied by human volunteers. In line with use of the device, the importance of child-protective, whilst the need for easy-to-open packaging was also stressed, as transdermal patches have been previously difficult to open [46]. Furthermore, suitable packaging presents an adherence aid opportunity as a reminder of application times, in replicating that of calendar packs employed with oral tablets.

Some unique concerns were raised exclusively within this study by this demographic. Interestingly, there was general apprehension of the inclusion of MN-mediated delivery into an already complicated drug regimen, particularly relevant for conditions in which exhibit polypharmacy, such as that of HIV. As it has been shown previously that increasing the number of tablets, frequency of dosing and complexity of therapy causes confusion and decreases adherence [47]. Therefore, this may present as a barrier to successful MN introduction to established therapy, at least initially, until numerous MN patches are available on the market for a variety of conditions. Further substantial concerns were expressed as to whether the novel concept MNs would be accepted as alternative to that of conventional oral ART, particularly for patients already established on well-tolerated therapy, providing suitable viral control. This reluctance to change is common to those either middle-aged or on established conventional therapy, as not only does the technology have to match the needs of the end-user, but the person needs to be convinced that the end benefits will be worth the costs to them, in terms of replacing their routine therapy [48]. Despite their apprehension, participants recognised the benefits that MN technology could potentially afford to their therapy and lifestyle, and expressed a willingness to try in the future, but highlighted that those of a younger generation or that are treatment naïve would make use of the greatest benefit of MN technology. As such, the diverse and varied suggestions made here regarding future use of MNs, reiterates the value of stakeholder engagement early in the development process and thus tailored treatment is required to meet the needs of the patient as an individual, not a condition in a collective sense.

### Phase two: questionnaire

The use of questionnaires provided some quantification of the overall consensus, or otherwise, on the views and opinions upon MN technology that had previously emerged through the phase one focus groups, discussion within the research team and existing literature [15, 16]. Thus, identifying whether or not the majority of respondents agreed or disagreed with the issues highlighted is possible. This mixed methods approach of gathering data from different background perspectives by various means increased the research validity through triangulation [49].

In terms of healthcare based research, a relatively high overall response rate of 34.7% was achieved (HCP, 23%; LP, 46%). The initial evaluation of the revealed respondents from both demographics recognised a relatively low knowledge of HIV, reporting that education surrounding HIV within Northern Ireland was somewhat limited. This is not unsurprising, and it has been previously demonstrated that low knowledge and misconceptions of HIV are commonplace in today’s society, [1] and reveals a growing need for increased awareness of HIV, as a lack of education ultimately leads to stigma resulting in poor public health outcomes [50]. Furthermore, public health interventions are more likely to be successfully introduced with high levels of public support [51]. Subsequently, with regard to the use of PrEP, despite members of the LP unfamiliarity with the preventative medication, both LP (> 90%) and HCP (> 85%) demonstrated favourable support of its prescribed use within Northern Ireland for high-risk individuals, and encourages its potential widespread implementation.

As no MN product has yet to reach market, it was expected that members of the LP would be relatively unfamiliar with the device (< 30%) in comparison with HCP (> 70%) who may have encountered MNs in literature. However, despite unfamiliarity with the technology, respondents from both demographics were capable of highlighting numerous potential benefits of the device, particularly in relation to hypodermic injection, with strong agreement stated for reduced pain on administration (62.5%), reduced fear (61.5%), reduced needle stick injuries (62.5%) and beneficial for needlephobic patients (61.1%). These main perceived benefits of MN technology in comparison with conventional hypodermic delivery, resonate with that of previous studies investigating end user perspectives of the technology [16]. Respondents also displayed optimism on the postulated self-application of the device, with 89.9% of HCP and 86.3% of LP agreeing they would be happy to use MNs themselves. In those expressing less confidence in their own ability, they then stated once they had been shown by either a HCP, clear instruction such as a PIL, or a positive feedback mechanism, they would then be happy to do so. The potential for self-use was further supported by respondents indicating they would like MNs for sale in pharmacies for at home use in the future, with 87% of HCPs and 91.4% of the LP indicating this choice, a much higher support than previous studies, thus demonstrating a growing confidence in the device [16]. However, despite an acknowledgement of the technology’s benefits, some potential concerns were also highlighted. Strangely, most concerns still indicated patient preference for conventional methods of delivery in certain circumstances, which was in line with the reluctance to change from established therapy expressed by Phase One focus group patients. However, it is interesting that the concerns highlighted by respondents in this study, were also raised by HCP and the LP in a study conducted by Birchall et al. [16] almost a decade ago, and despite scientific advancements of the technology addressing many of these issues since then, the same concerns were still expressed regardless. Thus, highlighting the need for greater public engagement to ultimately ensure MN acceptance.

Terminology surrounding MN technology has caused great debate in recent years for regulators and researchers alike [52], and now seemingly amongst end-users also. This was noticeably less so for questionnaire respondents, than that of the divided opinion expressed within the focus group discussion, as HCP and LP expressed more than 86 and 88%, respectively, in support of the name microneedles. In line with views expressed in the focus groups, the respondent minority whom disliked the name, once again highlighted this was due to inclusion of the term needles in the title. As such, this terminology must be considered carefully moving forward as patient acceptance of a medical device has been shown to be the greatest determinant of its success, or lack thereof [12].

Respondents demonstrated optimistic views upon diverse future clinical applications of the technology, which may lend to support of their accepted widespread use, with the greatest benefit envisaged for those unable to swallow medications, needlephobic patients and childhood vaccinations. Importantly, respondents recognised MN’s potential place within HIV, as 97.1% of HCPs and 98.6% of the LP stated support of their use. Furthermore, respondents indicated a strong preference of use MNs over traditional methods of delivery, namely that of hypodermic injection, suppository/pessary, transdermal patches or topical gels. However, it was clear that oral delivery, where available, remains the ideal route of choice from both demographics.

Both respondent demographics’ final overall impression of the technology was optimistic, with 97.1% of HCP and 98.6% of the LP reporting positive or strongly positive impressions. Encouragingly, for HCPs this is a much greater level of acceptance than that reported previously of 74% in a preliminary study a decade ago [16], demonstrating a growing support of their accepted use within clinical practice. Therefore, despite the many concerns raised, HCP and the LP appeared to welcome the potential value of this novel technology. Whilst these results may not be broadly generalisable, not one respondent was negative regarding the concept of MNs as a drug delivery approach; as such, this exploratory study displays great future promise for the successful implementation of MN as a widely accepted device by its intended end-users, and by those who administer them.

Moving forward in developing further translational research based on the findings from this mixed methods study, due consideration must be given to a number of factors when interpreting the results. As the nature of qualitative research, the findings are not broadly generalisable. Further to this, only two focus groups were conducted due to associated recruitment difficulties within this patient populating, as a result, data saturation could not be assumed to be achieved [53]. In addition, focus group demographics exhibited a strong male bias with a high mean age. Whilst this may be broadly representative of the population affected by HIV within this particular region (Northern Ireland), further follow up investigation involving a more diverse and younger population, potentially including more complex demographics that are pertinent to HIV such as, pregnant women, paediatrics and IV drug users, will now require exploration to expand upon this research. Thus, whilst it is not necessarily representative of the opinions of other patients with HIV however, the results are extremely valuable, and may be transferrable. In the latter stages of each study Phase, convenience and snowball recruitment approaches were employed, which may introduce a degree of selection bias, however it is an effective and economic strategy to reach potential participants [54]. Further consideration must be given that neither study Phase was designed to test the use of MNs, and additionally assumed a basic understanding of medical devices, particularly that of transdermal delivery, thus perceptions may be based on limited exposure to such technology. Consequently, an explanation of MN technology was provided at the beginning of every interaction due to the novelty of the topic and, although information relayed was of a factual nature only and MN visualisation was incorporated to facilitate the independent generation of ideas and opinions, this may in turn have affected the responses provided.

## Conclusion

This study provides the first insight to the views and opinions of patients living with HIV, HCPs and members of the LP, regarding the potential future use of MNs for drug delivery with a particular highlight on ARV delivery. Numerous benefits of the technology were recognised by demographics in both Phases of the study, particularly in relation to the potential for sustained release, enhanced adherence, self-application and as an alternative route of administration compared with current oral ART or that of the LA ARV injectable formulations under clinical investigation. Participants and respondents also identified relevant clinical applications for the use of MNs outside the scope of ARV delivery including delivery of the influenza vaccine or diagnostic fluid sampling. The terminology of the technology, presented conflicting opinions in both study Phases, while generally agreed that the name microneedles was an accurate descriptor of the device, in opposing cases there was a common sentiment that for successful translation from clinic to market, any reference to needle should be removed from the device due to negative connotations with that term. Participants further expressed conflicting views as to the preferred MN dosing schedule, which may indicate that in future development of MN systems, patch formulation of different duration may be required to suit the preference of the individual patient. The potential introduction of MN technology to replace current oral ART was highlighted by focus group participants, with treatment naïve patients postulated to avail the greatest benefit of such innovative MN technology, as those established on effective conventional therapies may express a reluctance to change, regardless of the benefits innovative MN technology may afford. Thus, patient education was revealed to be key to ensuring both acceptance and, additionally safe and effective use of MNs not only by those affected by HIV but also for general clinical applications. The role of HCPs was highlighted to be central to the success of such implementation further subsidised by significant marketing strategies to educate the general population relative to existing and widely accepted conventional delivery systems. The role of the HCP was further acknowledged in counselling and training of MN application, and that a positive feedback mechanism was generally required to ensure confidence in the correct technique. Further consideration must be afforded to the need for suitable packaging and potential mobile phone applications within the development process. Overall, respondents and participants in both study Phases were positive about the use of MN technology, displaying confidence that any concerns could be readily addressed early in future development efforts and relevant research to be conducted. Moving forward, it is clear that continued focused collaboration between academia, industry, HCPs and patients will be required to provide solutions and further improve this technology to ensure successful patient-centred MN commercialisation.
